# Respiratory Health Symptoms among Schoolchildren in Relation to Possible Food-Related Risk and Protective Factors

**DOI:** 10.3390/ijerph15030502

**Published:** 2018-03-13

**Authors:** Caradee Y. Wright, Vusumuzi Nkosi, Janine Wichmann

**Affiliations:** 1 Department of Geography, Geoinformatics and Meteorology, University of Pretoria, Pretoria 0001, South Africa; 2 Environment and Health Research Unit, South African Medical Research Council, Johannesburg 2094, South Africa; vusi.nkosi@mrc.ac.za; 3 School of Health Systems and Public Health, University of Pretoria, Pretoria 0001, South Africa; janine.wichmann@up.ac.za

**Keywords:** respiratory health symptoms, children, risk factors, air pollution, South Africa

## Abstract

Respiratory health outcomes are among the top five causes of child morbidity and mortality around the world. We aimed to investigate possible food-related risk and protective factors for respiratory health outcomes in children. Structured questionnaires completed by primary caregivers of 10-year old children were used to collect information on demographics, socio-economic status, house characteristics and child respiratory health status. Upper (URIs) and Lower (LRIs) respiratory illnesses comprised hay fever, and wheezing, asthma and bronchitis, respectively. Eight hundred questionnaires were distributed, 648 retrieved and 420 completed in full (52.5% response rate). The hay fever 6-month prevalence was 22.4% and wheezing had the highest 6-month prevalence among the LRIs (13.8%). The majority of children ate vegetables (75.5%), fruit (69.3%) and chicken or fish (81.7%) regularly. Nearly half of the children (45.5%) regularly ate processed food. Eating processed food regularly was statistical significantly associated with wheeze (Adjusted Odds Ratio (OR) = 2.65; 95% CI: 1.38–5.08), hay fever (OR = 1.62; 95% CI: 1.09–2.64) and bronchitis (OR = 1.27; 95% CI: 1.06–2.56). The study found an association between regular consumption of processed foods and wheeze, hay fever and bronchitis among 10 year old children. The regular consumption of processed food plays a role in adverse respiratory health effects among children and healthy eating is emphasized.

## 1. Introduction

Respiratory health and allergic reactions, for example, wheezing, asthma and hay fever, are a major cause of mortality and morbidity worldwide. Children are particularly susceptible. Acute respiratory infections, with symptoms such as coughing, phlegm, bronchitis, that is, some of the symptoms we investigated, account for an estimated 1.3 million deaths each year in children under 5 years of age, where 43% occur in Africa [1]. In South Africa, childhood respiratory infections are common and complicated by a high burden of HIV [2]. In Africa, asthma prevalence is said to have increased from 1990 to 2012 [3].

Risk factors associated with respiratory outcomes include overcrowding, poor sanitation and housing quality as well as exposure to environmental tobacco smoke (ETS) and indoor and outdoor air pollution [4]. Children are especially vulnerable to the hazardous respiratory effects of solid fuel use and the resultant indoor air pollution generated during cooking and heating indoors. Use of biomass fuels for cooking and heating is common in many parts of South Africa. Recently, the World Health Organization stated that electricity alone is the only recommended intervention to reduce indoor air pollution exposure in homes [5]. Other interventions, such as improved cook stoves or alternative lighting and burning techniques do not reduce particulate matter (PM) and other pollutant levels to exposure limits acceptable to remove risk to human health. Exposure to even low ambient outdoor air pollution has also been associated with various cause-specific mortality, including in Cape Town, South Africa [6]. Exposure to fine particulate matter and ozone increases the risk of respiratory infections in children, as well as affects immune function and causing inflammatory reactions [7].

Malnutrition, caused by both food insecurity and under-nourishment due to a lack of choice [8], is a particularly strong risk factor for the incidence of, and mortality due to, respiratory infections in children [4]. The dose-response function suggests that even relatively mild malnutrition increases respiratory health risks [9]. Flavonoids, plant metabolites known to provide health benefits through cell signaling pathways and antioxidant effects and specifically flavonoid supplementation, can decrease upper respiratory tract infection incidence by 33% (95% confidence interval: 31%, 36%) [10]. Hence, the importance of vegetables and fruits to protect against respiratory health has been emphasized. Some studies suggest that there is a benefit of antioxidant supplementation in moderating the effects of air pollution on lung function [10]. However, a lack of sufficient supporting evidence prevails and the need to eat healthily remains.

Regular consumption of processed foods and meat is associated with reduced lung function and subsequently several adverse respiratory health impacts [11]. Processed, or fast foods, are typically foods that are packaged in boxes, cans or bags post-processing and which may include preservatives, additives, artificial colorings and other chemical ingredients. Processed or ‘cured’ meat intake and the risk of chronic obstructive pulmonary disorder is related to the nitrites added to the meat products as preservatives and color fixatives [12]. These nitrites may amplify inflammatory processes in the airways among other negative effects. Here, we evaluate the respiratory health status of a sample of school-going children and investigate the association between food intake and respiratory health. The results will be useful in health promotion activities. As far as we know, this is the first study with a focus on food intake in Africa and which considered both indoor and outdoor air pollution exposure.

## 2. Methods

### 2.1. Sample

The study sample comprised 10-year old children from six, randomly-selected schools located in Gauteng province of South Africa. Ten year old children were selected since children at this age normally do not yet smoke actively and they are still developing physiologically [13]. All children of 10 years old, including disabled/handicapped children, in each of the six schools were invited to participate. Based on estimated asthma prevalence of 10% [14] among South Africa primary schoolchildren, a 5% margin of error and an estimate 80% response rate, the estimated sample size was 345. We assumed that at six schools, there would be on average 130 children aged 10 years and the study budget could cover the costs of questionnaire printing and transport to and from the six schools on multiple occasions to retrieve the completed questionnaires.

### 2.2. Procedures

A cross-sectional study was implemented and in 2010, school principals at each school were contacted telephonically to explain the reason for the study and the expected study outcomes. School principals completed an informed consent form and agreed to specific dates when questionnaires would be handed out to all 10-year-old children and sent home, together with informed consent forms, to their parent/guardian/caregiver to complete the questionnaire. The approximate number of 10-year-old children in each school and the language preferences of the children were provided by the principal. Researcher retrieval of completed questionnaires was planned 2 weeks thereafter.

A structured questionnaire served to collect data on demographics, socio-economic status, house characteristics as well as child health status. The questionnaires were translated into English, Afrikaans and Sesotho. There were 200 English, 200 Afrikaans and 400 Sesotho questionnaires printed. A pilot study was conducted in Gauteng Province (where researchers were based) to test the questionnaire. Notes were taken about items that were not clear or misinterpreted and questions were revised accordingly.

All data underwent double entry data capture into EpiData 3.1 (EpiData Association, Odense, Denmark). Permission for the study was obtained from the Departments of Education in Gauteng and Free State provinces where the schools were located. Research ethics clearance was granted by the Council for Scientific and Industrial Research Ethics Committee (REC number: 03/2010) and the University of Pretoria Ethics Committee (REC Number: S136/2010).

### 2.3. Questionnaire

The questionnaire was based on five questionnaires used previously, namely, the ATS-DLD-78-A questionnaire; the Canadian Air Quality and Health Study questionnaire (NHW/HPB-190-03040); the Harvard School of Public Health’s Children’s Health Study Questionnaire (NHW/HPB-190-03210); the 1990 Vaal Air Pollution Study questionnaire [15]; and a study on the respiratory health status of adults spending their developing years in a polluted area in South Africa [16].

The health questions focused on the child’s current health status related to lower and upper respiratory health outcomes: wheeze (does the child’s chest sound wheezy or make a whistling sound when he/she inhales/breathes in?), hay fever (parent-reported past 6 months), bronchitis (parent-reported past 6 months) and asthma (doctor-diagnosed ever). The prevalence of the hay fever and bronchitis was determined for the past six months.

The following questions provided information on possible risk factors related to diet. “Which of the following does the child eat regularly (at least three times a week, a conservative estimate adapted from the National Food Consumption Survey in South Africa [17]). The parent/guardian who completed the questionnaire could indicate “yes” or “no” for all food options, that is, (1) chicken or fish, (2) red meat, (3) processed food such as meat pies, polony, salami, fish fingers and so forth, (4) and fruits and (5) vegetables. Dietary intake as an exposure factor has been associated with respiratory disease outcomes [18].

Questions related to child demographics pertained to gender, home language, date of birth, current residence, time spent in the current town and where the child resided previously. Other questions provided information on household living conditions, also possible confounders: description of the type of home the child resides in, the number of bedrooms, the number of people in the home (<3 people/bedroom versus ≥3 people/bedroom), heating systems used in the home, use of electrical appliances, the presence of a fireplace and fuels used for cooking.

### 2.4. Data Analysis

Data were exported from EpiData 3.1 to STATA version 14 [19] and dependent variables were re-coded into binary variables. Respiratory illnesses were categorized into upper and lower respiratory tract illnesses. Upper respiratory illnesses (URIs) comprised hay fever. Lower respiratory illnesses (LRIs) comprised wheezing, asthma, and bronchitis. For clarity, asthma refers to ever being diagnosed with asthma by a medical doctor and not to asthma attacks or asthma symptoms, for example, wheezing.

Univariate and multiple logistic regression analyses (LRA) were conducted to estimate the crude and adjusted association between the various health outcomes, food types and possible confounders. Possible confounders included the following: Sex, ETS exposure at home, type of the residential home, fuel used for residential cooking, heating system used in the house and household crowdedness. The confounders were selected based on the logical assumption that they may be associated with both the health outcomes and the different food groups. Many of the selected confounders are related to socio-economic status, which influences the type of food consumed.

The associations are expressed as crude and adjusted odds ratios (ORs) along with their 95% confidence intervals (CI). Missing values were automatically excluded in each LRA model; therefore each multiple LRA model had a different sample size. To obtain adjusted ORs for the effect of eating processed food regularly on the health outcomes, both variables were placed in an initial LRA model. This was followed by the addition of a potential confounder in a stepwise manner starting with the most statistical significant from the univariate analysis. Each time a new potential confounder was added to the model, if the effect estimated between the health outcome and eating processed food regularly already in the model changed by 5%, then the additional variable was retained in the final multiple LRA, otherwise the variable was removed and a different one was added. At multiple LRA level, variables were deemed significant if *p*-values were <0.05. Regression analyses were not conducted for those health outcomes that had fewer than 20 study participants.

## 3. Results

### 3.1. Sample Description

Eight hundred questionnaires were distributed, 648 were retrieved and 420 were completed in full, providing a 52.5% response rate. Girls comprised 55.2% of the study population and boys 44.8% (Table 1). Several linguistic groups were present, that is, Sotho (55.6%), Afrikaans (15.8%), English (11.5%), Zulu (9.3%), Xhosa (3.8%), Swazi (0.24%) and other languages (3.6%) which were Venda, Pedi, Tswana and Tsonga. Our sample differed in language profile compared to that of the nearest large town of Vereeniging, where 34.8% spoke Afrikaans, 26.2% spoke Sesotho, 15.4% spoke English, 8.2% spoke Zulu and 4.1% spoke Xhosa [20]. The likely reason for this difference was that we had randomly selected more rural-based schools than urban schools. About 75.0% of the sample lived in single unattached households, 8.8% in single attached houses, 6.4% in flats and the remainder 3.8% in prefabricated homes. 

### 3.2. Prevalence of Allergies and Respiratory Infections

The 6-month prevalence of URIs and LRIs are shown in Table 2. Wheezing had the highest prevalence among the LRIs (13.8%).

### 3.3. Univariate Regression Analysis

Eating red meat regularly was a statistically significant risk factor for bronchitis (OR = 2.14; 95% CI: 1.12–4.11) (Table S1). Regularly eating meat and fruit were associated with asthma.

### 3.4. Multiple Regression Analysis

Eating processed food regularly was statistical significantly associated with wheeze (OR = 2.65; 95% CI: 1.38–5.08), hay fever (OR = 1.62; 95% CI: 1.09–2.64) and bronchitis (OR = 1.27; 95% CI: 1.06–2.56) (Table 3). Table S2 reports on the observed associations between the confounders and the health outcomes. Children that were exposed to ETS at home were 101% and 108% more likely to have wheeze and asthma, respectively (Table S2). Using wood/coal stove for heating the house was a significant risk factor for hay fever (OR = 2.91; 95% CI: 1.02–8.23. Eating red meat regularly increased the risk of having asthma and bronchitis. Household overcrowding was associated with increased risk for bronchitis (OR = 3.05; 95% CI: 1.30–7.17) and asthma (OR = 2.27; 95% CI: 1.93–5.51).

## 4. Discussion

The results of the respiratory health status of sampled 10-year old school-going children showed that less than a quarter of the sample were affected by URI hay fever (22.4%) and LRI wheeze (13.8%). Our asthma prevalence was 11.9% and was similar to that found among 7 and 8 year old South African children (living across multiple sites with varying air pollution levels) where the prevalence of reported asthma was 10.8% [14] as well as in the ISAAC study in Cape Town with an asthma prevalence of 13.3%. [21] In our study, apart from living in an area known to have high outdoor air pollution levels, the children were also exposed to indoor air pollution sources, most notably ETS exposure and polluting heating systems. Indoor sources of allergens were present, for example, pet ownership and mold. All of these factors contribute to the prevalence of respiratory health effects and as such, several were identified as risk factors for some of the respiratory health outcomes in this study. 

We set out to investigate the association between food intake and respiratory health outcomes. It was encouraging to note how many children regularly ate healthily; with more than two-thirds of the children eating fruits (69.3%), vegetables (75.5%) and chicken (81.7%). We found chicken and/or fish as well as fruits consumption was protective against bronchitis. However, there is generally a lack of sufficient evidence that supports the protective effect of fruit and vegetables on respiratory health effects [22].

Our questionnaire item combined chicken and/or fish consumption thereby making it difficult to isolate which item the respondent was referring to in their response. The consumption of foods rich in n-3 polyunsaturated fatty acids (PUFA) has been proposed to protect against childhood asthma and fish intake was associated with a lower prevalence of asthma among non-atopic children (OR = 0.61; CI: 0.43–0.87) [23]. Current evidence indicates that fish intake in infancy could reduce the risk of eczema and allergic rhinitis in children, whereas maternal fish intake during pregnancy does not affect any atopic outcome [24]. The intake of fish per se in infancy, not specially n-3 LC-PUFAs (n-3 long-chain polyunsaturated fatty acids), may have an allergy protective effect. High-quality and adequately powered randomized controlled trials are warranted to confirm this. It is not clear whether fish oil can be used to treat children with asthma as the two studies conducted to date give divergent results. Further studies of increased long-chain n-3 PUFA provision in during pregnancy, lactation and infancy are needed to more clearly identify the immunologic and clinical effects in infants and children and to identify protective and therapeutic effects and their persistence [25]. 

More than half (54.0%) of the children in this study regularly ate red meat. We found red meat to be a significant risk factor for asthma and bronchitis. McKeever et al. [26] did a study on the patterns of dietary intake in relation to respiratory disease and a more traditional diet, that is, a high intake of meat and potatoes. They found that such a diet was associated with a lower forced expiratory volume, whereas a more cosmopolitan diet was associated with an increased risk of wheezing and asthma but not bronchitis [26].

We found that nearly half of the children sampled (45.5%) regularly ate processed food. The regular consumption of processed foods, such as crumbed, boxed and frozen fish and chicken, was a risk factor for three respiratory health outcomes: URI hay fever and LRIs wheeze and bronchitis. We were not able to find any other studies supporting an association between eating processed food and hay fever, although one review did not find widespread or consistent links between mothers’ dietary intake and atopic outcomes in their children [27]. Eating processed food regularly was identified as a significant risk factor for wheeze. This is consistent with findings from studies conducted in the Mediterranean, [28] Canada, [29] Colombia [30] and India [31].

The temporal relationship between dietary intake and respiratory health outcomes is critical in determining causality as most associations between diet and respiratory health outcomes are drawn from cross-sectional studies. Information though is thus limited as to whether dietary intake is truly involved in the development of certain respiratory health outcomes. Data on induction time or reversibility of the potential effect of diet on these health outcomes is scarce and requires further investigation [32,33].

Children’s respiratory health status and the exposure, both risk and protective factors, were assessed at the same point in time. This is an inherent limitation of all cross-sectional studies. The presence of systematic error (bias) and random error may influence the validity (accuracy) and precision (reliability) of our study results, respectively. Observer bias may be low as the parents/guardians/caregivers who completed the questionnaires may not have had any prior knowledge of possible association between the health outcomes and possible risk and protective factors and the questionnaire that was used during fieldwork was based on the five previously used questionnaires. The school study sample was randomly selected with the aid of a biostatistician; hence selection bias may be low. We defined ‘regular consumption’ as at least three times a week, a conservative estimate adapted from the National Food Consumption Survey in South Africa and this may have influenced our results. Recall bias on the food intake and cofounder variables may be low based on the wording used in the questionnaire, for example, during the past 2 weeks. Except for asthma (recorded as doctor-diagnosed ever), we used parent-reported health outcomes from the past 6 months and this may have introduced recall bias. Reporting bias may have influenced parental response. While it would have been preferred that parent-reported health outcomes of their child were confirmed by doctor-diagnosis, this was not possible in the study. Our findings among 10-year old children may not be relevant for children of other ages whose diet differs from the children sampled in this study. The food intake at 10 years of age was assumed to be similar to diet throughout childhood. This assumption may introduce biological variability (i.e., random error) which may weaken chance of finding a significant association with the health outcomes. Random error introduced due to measurement variability may be low seeing that the data were entered twice. Random error introduced due to sampling variability may be high seeing that data of only 420 completed questionnaires were eligible for inclusion in the study (52.5% response rate).

## 5. Conclusions

The study found an association between regular consumption of processed foods and wheeze, hay fever and bronchitis among 10 year old children and serves as a suitable baseline for assessing future trends in the prevalence of wheeze, hay fever and bronchitis in similar settings. The results will also be useful in health promotion activities.

## Figures and Tables

**Table 1:** Demographic variables and living conditions of the sample population.

Characteristic	Response Items	Number of Children	Percentage of Children
		(n)	(%)
Eating chicken or fish regularly	Yes	343	81.7
	No	77	18.3
Eating red meat regularly	Yes	193	54.0
	No	227	46.0
Eating fruits regularly	Yes	291	69.3
	No	129	30.7
Eating vegetables regularly	Yes	317	75.5
	No	103	24.5
Eating processed food	Yes	191	45.5
	No	229	54.5
Sex	Boys	188	44.8
	Girls	232	55.2
Type of home	Single, not attached	315	75.0
	Single, attached	37	8.8
	Flat	27	6.4
	Pre-fabricated	25	6.0
	*Missing*	*16*	*3.8*
ETS exposure at home	Yes	93	22.1
	No	320	76.2
	*Missing*	*7*	*1.7*
Fuel used for cooking	Electricity	392	93.3
	Gas	14	3.3
	Paraffin	4	1.0
	Wood	3	0.7
	Coal	2	0.5
	*Missing*	*5*	*1.2*
Heating system used at home	Fireplace	227	54.1
	Gas/Paraffin stove	169	40.2
	Wood/Coal stove	18	4.3
	Asbestos heater	6	1.4
Household crowding (people/room)	<3	166	39.5
	≥3	254	60.5

**Table 2:** Prevalence of respiratory symptoms, diseases and allergies among children.

Outcome	Response Item	Number of Children (n)	Percentage of Children (%)
**Wheeze**	Yes	58	13.8
	No	347	82.6
	Total	405	
**Hay fever**	Yes	94	22.4
	No	326	77.6
	Total	420	
**Bronchitis**	Yes	43	10.2
	No	377	89.8
	Total	420	
**Asthma**	Yes	50	11.9
	No	366	87.1
	Total	416	

**Table 3:** Unadjusted and adjusted odds ratios of respiratory symptoms, diseases and allergies with eating processed food regularly among children.

Health Outcomes	Unadjusted OR	(95% CI)	*p*-Value	Adjusted OR	(95% CI)	*p*-Value
**Wheeze ^a^**	2.60	1.46–4.68	0.001	2.65	1.38–5.08	0.003
**Hay fever ^b^**	1.58	0.99–2.50	0.053	1.62	1.09–2.64	0.041
**Asthma ^c^**	1.12	0.62–2.03	0.698	0.83	0.43–1.58	0.561
**Bronchitis ^d^**	1.16	0.62–2.19	0.641	1.27	1.06–2.56	0.040

Notes: Children that were not eating processed food regularly were used as reference category. ^a^ Model adjusted for environmental tobacco smoke (ETS) exposure at home, eating chicken and/or fish regularly, eating red meat regularly, eating vegetables regularly, eating fruit regularly, type of heating system used in the house, type of home residing in and household overcrowding but not for sex and type of fuel used for cooking at home. ^b^ Model adjusted for ETS exposure at home, eating red meat regularly, eating vegetables regularly, type of heating system used in the house, type of home residing in and household overcrowding but not for sex, type of fuel used for cooking at home, eating fruit regularly, eating chicken and/or fish regularly. ^c^ Model adjusted for ETS exposure at home, eating chicken and/or fish regularly, eating red meat regularly, eating vegetables regularly, eating fruit regularly, type of heating system used in the house type of home residing in and household overcrowding but not for sex and type of fuel used for cooking at home. ^d^ Model adjusted for ETS exposure at home, eating chicken and/or fish regularly, eating red meat regularly, eating vegetables regularly, eating fruit regularly, household overcrowding, type of house residing in, type of heating system used in the house but not for type of fuel used for cooking at home and sex.

## Supplementary Materials

The following are available online at http://www.mdpi.com/1660-4601/15/3/502/s1, Table S1: Unadjusted odds ratios of respiratory symptoms, diseases and allergies among children, Table S2: Adjusted odds ratios of respiratory symptoms, diseases and allergies among children.

## Abbreviations

LC-PUFAs	n-3 long-chain polyunsaturated fatty acids
LRA	Logistic Regression Analysis
LRI	Lower Respiratory Tract Infection
URTI	Upper Respiratory Tract Infection
ETS	Environmental Tobacco Smoke
OR	Odds Ratio
CI	Confidence Interval
ISAAC	The International Study of Asthma and Allergies in Childhood
IgE	immunoglobulin E
PM	Particulate Matter
