# Supplementary material for: Respiratory Health Symptoms among Schoolchildren in Relation to Possible Food-Related Risk and Protective Factors
Source: Int J Environ Res Public Health. 2018 Mar 13;15(3):502. doi: 10.3390/ijerph15030502 (PMC5877047; doi:10.3390/ijerph15030502)
Supplement: Supplementary file 1 [file ijerph-15-00502-s001.pdf]

# Respiratory health symptoms among schoolchildren in relation to possible food-related risk and protective factors

**Table S1.** Unadjusted odds ratios of respiratory symptoms, diseases and allergies among children where * indicates the reference group.

| Outcome | Risk factor | Crude Odds Ratio | Confidence Interval (95% CI) | p-value |
|---|---|---|---|---|
| Wheeze | Eating chicken and/or fish regularly | 1.30 | 0.61-2.78 | 0.495 |
| | Eating red meat regularly | 0.57 | 0.32-1.02 | 0.061 |
| | Eating fruit regularly | 1.69 | 0.88-3.27 | 0.116 |
| | Eating vegetables regularly | 1.47 | 0.73-2.97 | 0.277 |
| | Sex | | | |
| | *Girls** | 1.00 | | |
| | *Boys* | 1.25 | 0.72-2.19 | 0.427 |
| | Type of house residing in | | | |
| | *Single family house not attached** | 1.00 | | |
| | *Single family house attached* | 0.30 | 0.70-1.31 | 0.110 |
| | *Flat* | 0.48 | 0.11-2.13 | 0.338 |
| | *Prefabricated house* | 1.07 | 0.34-3.25 | 0.910 |
| | ETS exposure at home | 1.71 | 0.93-3.16 | 0.087 |
| | Fuel used for cooking at home | | | |
| | *Electricity** | 1.00 | | |
| | *Gas* | 1.00 | 0.22-4.59 | 1.00 |
| | *Paraffin* | 3.00 | 0.28-33.66 | 0.373 |
| | *Wood* | - | - | - |
| | *Coal* | 6.00 | 0.37-97.37 | 0.208 |
| | Heating system used in the house | | | |
| | *Fireplace** | 1.00 | | |
| | *Gas/Paraffin heater* | 0.72 | 0.40-1.31 | 0.283 |
| | *Wood/coal stove* | 0.71 | 0.16-3.25 | 0.662 |
| | *Asbestos heater* | 2.67 | 0.47-15.15 | 0.267 |
| | Household overcrowded | | | |
| | *< 3** | 1.00 | | |
| | *≥ 3* | 0.69 | 0.39-1.20 | 0.190 |
| Hay fever | Eating chicken and/or fish regularly | 0.92 | 0.97-3.80 | 0.063 |
| | Eating red meat regularly | 0.99 | 0.62-1.57 | 0.963 |
| | Eating fruit regularly | 1.60 | 0.94-2.72 | 0.083 |
| | Eating vegetables regularly | 0.93 | 0.55-1.58 | 0.797 |
| | Sex | | | |
| | *Girls** | 1 | | |
| | *Boys* | 0.99 | 0.63-1.58 | 0.986 |
| | Type of house residing in | | | |
| | *Single family house not attached** | 1 | | |
| | *Single family house attached* | 0.62 | 0.24-1.54 | 0.303 |
| | *Flat* | 0.40 | 0.11-1.36 | 0.144 |
| | *Prefabricated house* | 1.24 | 0.50-3.09 | 0.638 |
| | ETS exposure at home | 1.00 | 0.58-1.75 | 0.987 |
| | Fuel used for cooking at home | | | |
| | *Electricity** | 1.00 | | |
| | *Gas* | 0.92 | 0.25-3.35 | 0.193 |
| | *Paraffin* | - | - | - |
| | *Wood* | 1.68 | 0.15-18.72 | 0.674 |

| | | | | |
|---|---|---|---|---|
| | *Coal* | - | - | - |
| | Heating system used in the house | | | |
| | *Fireplace** | 1.00 | | |
| | *Gas/Paraffin heater* | 1.11 | 0.69-1.80 | 0.670 |
| | *Wood/coal stove* | 2.44 | 0.90-6.62 | 0.081 |
| | *Asbestos heater* | 1.91 | 0.34-10.77 | 0.461 |
| | Household overcrowded | | | |
| | *< 3** | 1.00 | | |
| | *≥ 3* | 0.95 | 0.60-1.52 | 0.839 |
| Bronchitis | Eating chicken and/or fish regularly | 0.61 | 0.30-1.29 | 0.198 |
| | Eating red meat regularly | 2.14 | 1.12-4.11 | 0.022 |
| | Eating fruit regularly | 0.72 | 0.37-1.39 | 0.331 |
| | Eating vegetables regularly | 0.72 | 0.36-1.45 | 0.360 |
| | Sex | | | |
| | *Girls** | 1.00 | | |
| | *Boys* | 1.20 | 0.64-2.26 | 0.571 |
| | Type of house residing in | | | |
| | *Single family house not attached** | 1.00 | | |
| | *Single family house attached* | 0.42 | 0.96-1.80 | 0.241 |
| | *Flat* | 0.58 | 0.13-2.56 | 0.475 |
| | *Prefabricated house* | - | - | - |
| | ETS exposure at home | 0.53 | 0.22-1.29 | 0.162 |
| | Fuel used for cooking at home | | | |
| | *Electricity** | 1.00 | | |
| | *Gas* | 0.64 | 0.08-5.02 | 0.672 |
| | *Paraffin* | - | - | - |
| | *Wood* | - | - | - |
| | *Coal* | - | - | - |
| | Heating system used in the house | | | |
| | *Fireplace** | 1.00 | | |
| | *Gas/Paraffin heater* | 2.04 | 1.06-3.94 | 0.033 |
| | *Wood/coal stove* | 1.54 | 0.33-7.28 | 0.583 |
| | *Asbestos heater* | - | - | - |
| | Household overcrowded | | | |
| | *< 3** | 1.00 | | |
| | *≥ 3* | 3.16 | 1.43-6.99 | 0.005 |
| Asthma | Eating chicken and/or fish regularly | 1.04 | 0.48-2.24 | 0.921 |
| | Eating red meat regularly | 3.14 | 1.66-5.95 | <0.001 |
| | Eating fruit regularly | 3.03 | 1.32-6.94 | 0.009 |
| | Eating vegetables regularly | 1.81 | 0.82-4.00 | 0.140 |
| | Sex | | | |
| | *Girls** | 1.00 | | |
| | *Boys* | 1.51 | 0.84-2.74 | 0.172 |
| | Type of house residing in | | | |
| | *Single family house not attached** | 1.00 | | |
| | *Single family house attached* | 0.54 | 0.16-1.83 | 0.322 |
| | *Flat* | 0.25 | 0.03-1.93 | 0.186 |
| | *Prefabricated house* | 0.25 | 0.04-1.93 | 0.186 |
| | ETS exposure at home | 1.72 | 0.90-3.29 | 0.098 |
| | Fuel used for cooking at home | | | |
| | *Electricity** | 1.00 | | |
| | *Gas* | 1.98 | 0.53-7.35 | 0.308 |
| | *Paraffin* | - | - | - |
| | *Wood* | - | - | - |
| | *Coal* | - | - | - |
| | Heating system used in the house | | | |

| | | | | |
|---|---|---|---|---|
| | *Fireplace** | 1.00 | | |
| | *Gas/Paraffin heater* | 1.79 | 0.96-3.35 | 0.068 |
| | *Wood/coal stove* | 4.27 | 1.37-13.35 | 0.013 |
| | *Asbestos heater* | - | - | - |
| | Household overcrowded | | | |
| | *< 3** | 1.00 | | |
| | *≥ 3* | 3.81 | 1.74-8.35 | 0.001 |

**Table S2.** Adjusted odds ratios of respiratory symptoms, diseases and allergies among children where ″*″ indicates the reference group.

| Outcome | Risk factor | Crude Odds Ratio | Confidence Interval (95% CI) | p-value |
|---|---|---|---|---|
| Wheeze | Eating chicken and/or fish regularly | 0.93 | 0.36-2.40 | 0.888 |
| | Eating red meat regularly | 0.39 | 0.20-0.77 | 0.007 |
| | Eating fruit regularly | 1.10 | 0.44-2.80 | 0.834 |
| | Eating vegetables regularly | 1.40 | 0.57-3.43 | 0.464 |
| | Type of house residing in | | | |
| | *Single family house not attached** | 1.00 | | |
| | *Single family house attached* | 0.24 | 0.05-1.23 | 0.087 |
| | *Flat* | 0.36 | 0.08-1.79 | 0.215 |
| | *Prefabricated house* | 0.89 | 0.27-3.01 | 0.858 |
| | ETS exposure at home | 2.01 | 1.02-4.00 | 0.044 |
| | Heating system used in the house | | | |
| | *Fireplace** | 1.00 | | |
| | *Gas/Paraffin heater* | 0.72 | 0.04-1.49 | 0.678 |
| | *Wood/coal stove* | 0.96 | 0.16-4.62 | 0.394 |
| | *Asbestos heater* | 3.64 | 0.55-24.16 | 0.181 |
| | Household overcrowded | | | |
| | *< 3** | 1.00 | | |
| | *≥ 3* | 0.69 | 0.35-1.36 | 0.287 |
| Hay fever | Eating red meat regularly | 0.88 | 0.52-1.49 | 0.635 |
| | Eating vegetables regularly | 0.82 | 0.46-1.48 | 0.525 |
| | Type of house residing in | | | |
| | *Single family house not attached** | 1.00 | | |
| | *Single family house attached* | 0.63 | 0.15-1.56 | 0.349 |
| | *Flat* | 0.35 | 0.60-4.59 | 0.108 |
| | *Prefabricated house* | 1.19 | 0.42-2.29 | 0.730 |
| | ETS exposure at home | 1.03 | 0.58-1.85 | 0.895 |
| | Heating system used in the house | | | |
| | *Fireplace** | 1.00 | | |
| | *Gas/Paraffin heater* | 1.01 | 0.67-1.82 | 0.705 |
| | *Wood/coal stove* | 2.91 | 1.02-8.29 | 0.046 |
| | *Asbestos heater* | 1.90 | 0.32-11.08 | 0.222 |
| | Household overcrowded | | | |
| | *< 3** | 1.00 | | |
| | *≥ 3* | 0.79 | 0.45-1.37 | 0.349 |
| Bronchitis | Eating chicken and/or fish regularly | 0.55 | 0.24-1.26 | 0.155 |
| | Eating red meat regularly | 2.49 | 1.14-5.45 | 0.022 |
| | Eating fruit regularly | 0.42 | 0.16-1.03 | 0.059 |
| | Eating vegetables regularly | 0.76 | 0.32-1.84 | 0.547 |
| | Type of house residing in | | | |
| | *Single family house not attached** | 1.00 | | |
| | *Single family house attached* | 0.35 | 0.72-1.72 | 0.198 |

| | | | | |
|---|---|---|---|---|
| | *Flat* | 0.93 | 0.19-4.68 | 0.934 |
| | *Prefabricated house* | - | - | - |
| | ETS exposure at home | 0.50 | 0.19-1.28 | 0.149 |
| | Heating system used in the house | | | |
| | *Fireplace** | 1.00 | | |
| | *Gas/Paraffin heater* | 1.86 | 0.93-3.70 | 0.079 |
| | *Wood/coal stove* | 0.99 | 0.19-5.00 | 0.989 |
| | *Asbestos heater* | - | - | - |
| | Household overcrowded | | | |
| | *< 3** | 1.00 | | |
| | *≥ 3* | 3.05 | 1.30-7.17 | 0.011 |
| Asthma | Eating chicken and/or fish regularly | 0.71 | 0.29-1.74 | 0.451 |
| | Eating red meat regularly | 2.02 | 1.98-4.14 | 0.044 |
| | Eating fruit regularly | 2.11 | 0.73-6.13 | 0.169 |
| | Eating vegetables regularly | 0.81 | 0.30-2.16 | 0.674 |
| | Type of house residing in | | | |
| | *Single family house not attached** | 1.00 | | |
| | *Single family house attached* | 0.94 | 0.24-3.70 | 0.932 |
| | *Flat* | 0.52 | 0.06-4.32 | 0.548 |
| | *Prefabricated house* | 0.61 | 0.07-5.23 | 0.656 |
| | ETS exposure at home | 2.08 | 1.03-4.20 | 0.040 |
| | Heating system used in the house | | | |
| | *Fireplace** | 1.00 | | |
| | *Gas/Paraffin heater* | 1.77 | 0.90-3.46 | 0.096 |
| | *Wood/coal stove* | 2.13 | 0.63-7.16 | 0.218 |
| | *Asbestos heater* | - | - | - |
| | Household overcrowded | | | |
| | *< 3** | 1.00 | | |
| | *≥ 3* | 2.27 | 1.93-5.51 | 0.005 |
